# Evaluation of Deformation for Steel Fiber Concrete Beams with BFRP Tendons Eroded by Seawater under Cyclic Loading

**DOI:** 10.3390/polym15010062

**Published:** 2022-12-23

**Authors:** Haitang Zhu, Qun Chen, Zongze Li, Yin Zhang, Wencheng Duan, Zemin Li

**Affiliations:** 1 School of Civil Engineering, Henan University of Engineering, Zhengzhou 451191, China; 2 School of Water Resources and Civil Engineering, Zhengzhou University, Zhengzhou 450001, China

**Keywords:** cyclic loading, deformation, BFRP-SFRC beams, seawater erosion

## Abstract

Steel fiber-reinforced concrete (SFRC) beams with fiber-reinforced polymer (FRP) bars are promising new composite structures. To investigate the durability of BFRP-SFRC beams, eleven beams were fabricated and conducted via four-point bending tests under cyclic loading. The experimental variables included BFRP reinforcement ratios, pre-cracked widths and environments (Natural or Seawater erosion). Experiment results revealed that the load–deflection curves of BFRP-SFRC beams showed bilinear growth. With the increase in loading and unloading cycles, the peak load and energy consumption of the tested beams decreased, and the impact of loading and unloading cycles on the flexural performances of the BFRP-SFRC beams enhanced with the increase in displacement. Under the same load, as the pre-crack width increases, the deflection of the BFRP-SFRC beam decreases. The deflection of the beam with a pre-crack width of 0.4 mm was 1.34 times than that of the beam without a pre-crack at the load of 100 kN. What is more, the pre-crack width had a bad effect on the energy consumed by the BFRP-SFRC beams. Compared with no pre-crack beam, the energy consumed by the beams with 0.02, 0.2 and 0.4 mm pre-crack width were decreased by 1.5%, 7.8% and 11.0% at the 18 mm displacement, respectively. Significantly, the effect of sea water erosion on the energy consumption of tested beams with high BFRP reinforcement ratios were smaller than that of tested beams with low BFRP reinforcement ratios. Finally, a calculation model of deformation of BFRP-SFRC beams under seawater erosion environments was proposed based on the effective moment of inertia methods. Compared with the existing calculation methods, this model was better correlated with the experimental results.

## 1. Introduction

In recent years, with the continuous exploration of marine resources, reinforced concrete (RC) structures have been widely used in marine constructions, such as seawalls, ports, sea-crossing bridges, undersea tunnels and offshore platforms. However, the chloride ions in the seawater quickly corroded the traditional RC structures, which had become the main reason for affecting the durability of the RC structures in marine engineering [1]. The use of fiber-reinforced polymer (FRP) bars with excellent corrosion resistance instead of steel bars in concrete structures can fundamentally solve the corrosion problem of steel bars [2]. In addition, FRP bars also are light weight, high strength, low creep, non-conductive and have stable chemical properties, which will be conducive to the promotion and application of FRP bars in harsh environments [3].

Previous studies have shown that FRP bars have excellent corrosion resistance in the short term, but the mechanical properties after immersion in seawater for a long time decrease to a certain extent [4,5,6,7,8,9,10]. Studies [4,6] have shown that the main factors affecting the durability of FRP bars include ambient temperature, pH values and resin type. According to studies of the mechanical properties of BFRP bars at different temperatures and immersion environments under marine erosion, Lv et al. [7] found that the marine environment had little effect on the elastic model, but the tensile strength was reduced by 10%. Similar findings were also reported by other researchers [8]. Duo et al. [11] collected 557 pieces of tensile strength and elastic modulus test data of GFRP and BFRP bars in different harsh environments, and found that GFRP and BFRP bars degraded fastest in an alkali environment, followed by a water environment, followed by an acid environment, and lowest in a salt environment. The degradation rate of BFRP bars was faster than that of GFRP bars under the same environment. Azzam Ahmed [12] reviewed the durability of FRP bars, and the results showed that the durability of FRP bars would be affected by the type of FRP bars and the surface protection of the bars and concrete. The research mainly focused on the application of GFRP bars in the marine environment, while there was less research on BFRP bars. The author believed that more research on simulation environments was needed in the future to fill the knowledge gap of the long-term performance of BFRP bars in high-performance concrete under continuous loads and corrosive solutions in coastal and marine environments. In summary, the tensile properties of FRP bars were significantly reduced under long-term marine environment erosion, which made its structural properties deteriorate. Some scholars [7,13] found through studying the long-term performance of FRP-reinforced concrete structures in marine environments that sea water corrosion reduced the ultimate bearing capacity and durability of the structures. However, relatively few articles on the sea water erosion of BFRP bars reinforced-concrete structures have been studied, so it is of great significance to study the long-term flexural performances of BFRP reinforced-concrete structures in the marine environment.

Different from RC structures’ performances, due to the low elastic modulus and linear elastic brittle behaviors of FRP bars, the FRP-reinforced concrete structures have large deflection and poor ductility [14]. Some of the literature [15,16,17] show that the incorporation of fibers in concrete was an effective way to solve the large deflection and poor ductility of FRP-reinforced concrete beams. In addition, the loads of marine structural buildings are mostly repeated loads. Zhu et al. [18] found that the load of FRP-reinforced SFRC beams were reduced by 10 % after three repeated cycles of loading and unloading. In summary, the existing literature has mainly studied the durability of FRP bars [19,20], and some studies have paid attention to the flexural performances of FRP-reinforced SFRC under long-term seawater immersion. In particular, the bending properties of SFRC beams reinforced with BFRP bars (BFRP-SFRC beams) after long-term seawater erosion have rarely been studied. Therefore, the study of the flexural performances of FRP-reinforced SFRC beams under repeated loads will provide effective data and theoretical support for similar practical projects.

Above all, this paper conducted a study of the flexural properties of BFRP-reinforced SFRC under long-term seawater corrosion. In this research, BFRP-SFRC beams were prepared and immersed in a simulated seawater solution for 365 d. A four-point bending test was carried out on eleven beams. The effects of BFRP reinforcement ratios, pre-cracked widths and environments on characteristic loads, load–displacement curves, envelope curves, residual deflections and energy consumption capacity were investigated. Finally, a new deflection calculation model for the eroded BFRP-SFRC beams was established based on the effective moment of inertia method. Compared with other models, this model was closer to the experimental results.

## 2. Materials and Methods

### 2.1. Materials Properties

#### 2.1.1. Concrete

**Figure 1:**
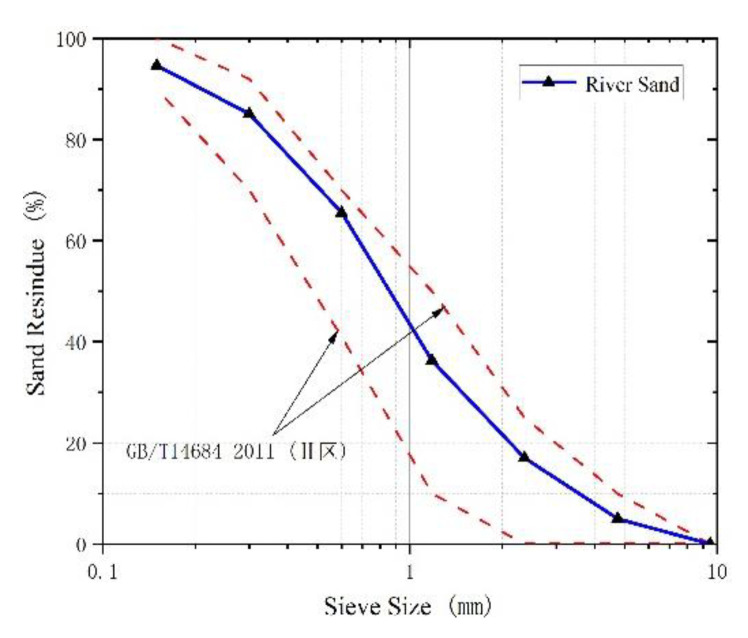
Grading curve.

Natural crushed stone with 5 to 20 mm particle sizes was used as coarse aggregate. And choose natural river sand as fine aggregate, whose fineness modulus was 2.59. Figure 1 presents the grading curve of fine aggregate and the limits (upside and downside) of Chinese standard GB/T14684-2011 [21]. The slump of the concrete matrix should be greater than 50 mm, which was to make the steel fiber evenly distributed in the concrete matrix. The water-reducers (produced by Sobute New Materials Co., Ltd. (Nanjing, China) were used in the process of preparing concrete. Based on the requirements of Chinese standard GB175-2007 [22] for cement, Portland cement (42.5 R) was utilized as a cementitious material in pouring concrete, which was produced by Henan Mengdian Group Cement Co., Ltd. (Xinxiang City, China).

**Figure 2:**
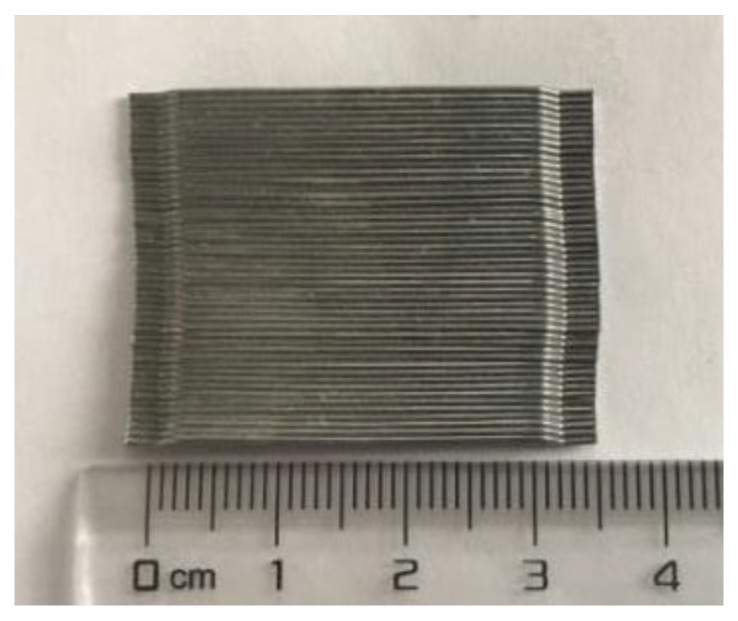
Hooked steel fiber.

Hooked steel fibers with a diameter of 0.55 mm and a length of 35 mm were used as reinforcement materials in the concrete matrix, which was produced by Bekaert Applied Materials Technology Corporation. Figure 2 shows the shape of the hooked steel fiber. Adding a steel fiber volume fraction of 1% can improve the ductility of BFRP-RC beams by 277.8% [23]. So, the steel fiber volume fraction of 1% was chosen in this paper. According to JG/T 472–2015 [24], the mix proportion of concrete was designed, as shown in Table 1. The specimens were labeled as ”BN”, “CN”, “E” or “N”, where “BN” referred to the BFRP reinforcement ratios, ”CN” represented pre-cracked widths, and “N” or “E” denoted the environment of the beam during one year (Natural or Simulated seawater solution erosion). For instance, ‘B0.56C0E’ referred to the beam with a BFRP reinforcement ratio of 0.56%, pre-cracked width of 0 mm, and the specimens were immersed in simulated seawater solution.

**Figure 3:**
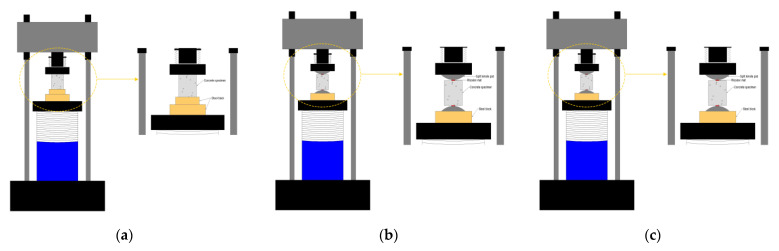
Testing method for mechanical properties of concrete, (**a**) compression test; (**b**) splitting tensile test; (**c**) Test of elastic modulus.

To obtain the basic mechanical properties of the beams, six 150 × 150 × 150 mm^3^ concrete cubes and six 150 × 150 × 300 mm^3^ concrete prisms were cast and tested. The test methods of Chinese standard GB/T 50081-2019 [25] were adopted for the compressive strength and splitting tensile strength. The loading rates of compressive strength and splitting tensile strength were 0.5 and 0.05 MPa/s, respectively. The acquisition system recorded the peak load during the test. A schematic diagram of compression and splitting tensile strength tests is shown in Figure 3.

The compressive strength, *f_cc_* (MPa), can be calculated by Equation (1):

(1) $$f_{\mathrm{cc}}=\frac{P}{A},$$

where *P* (N) is the peak load; *A* (mm2) is the pressure area of the specimen.

The split tensile strength, *f*_ts_ (MPa), which can be calculated by Equation (2):

(2) $$f_{\mathrm{ts}}=\frac{2P}{\pi A}=0.637\frac{P}{A},$$

Elastic modulus is an important performance parameter of engineering materials, which specific values were measured according to the Chinese Standard GB/T 50081-2019 [25]. *E_c_* is calculated by Equation (3):

(3) $$E_{c}=\frac{P_{a}-P_{0}}{A}\times \frac{L}{\Delta_{n}},$$

where *E_c_* (Mpa) is the elastic modulus of concrete; *P_a_* (N) is the load value when the stress is one-third of the axial compressive strength; *P*_0_ (N) is the initial load when the stress is 0.5 MPa; *A* (mm2) is the pressure area of the specimen; *L* is the current gauge length of the test specimen, which is 150 mm in this paper; $\Delta_{n}=\varepsilon_{a}-\varepsilon_{0}$ is the average deformation of the specimen from *P_a_* to *P*_0_.

#### 2.1.2. FRP Bars

The main longitudinal reinforcement materials selected were basalt fiber reinforced polymer with 12 mm and 14 mm diameters, which were produced by Jiangsu Green Valley New Material Technology Development Co., Ltd. (Nanjing, China). The mechanical properties of BFRP bars were tested according to ACI 440.3R-12 [26]. BFRP bars were tested in linear elastic behavior before rupture. The tensile strength and elastic modulus of the BFRP bars before and after seawater erosion (with the average of 5 samples taken as the relevant representative value) are shown in Table 2.

#### 2.1.3. Simulated Seawater Solution

**Figure 4:**
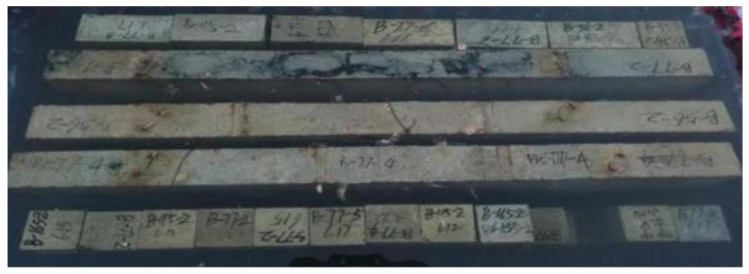
Simulated seawater immersion.

The average salt content in the sea area around China was approximately 3.5%. Therefore, the average salt concentration of the simulated seawater solution in this test was 3.5%, and the content of each component is shown in Table 3. Theerosion time was 365 d. The concrete cubes and test beams were soaked under the same conditions, as shown in Figure 4.

### 2.2. Specimen Design

The experimental program consists of 11 BFRP-SFRC beams, with four beams in natural environment (series 1) and seven beams after seawater erosion for 365 days (series 2). In order to study the mechanical performances of BFRP-SFRC beams with initial cracks after immersion in seawater for one year, three beams were loaded until the pre-cracked widths were 0.02, 0.2 and 0.4, respectively, before immersion. The cross-section dimensions of all beams were 150 mm × 300 mm and 2100 mm long.

Since the failure mode of a FRP fracture is sudden and catastrophic, FRP-RC structures are usually designed for concrete crushing failure. According to ACI 440. 1R-15 [27], the balanced reinforcement ratio of FRP-RC beams can be computed from Equation (4):

(4) $$\rho_{fb}=0.85\beta_{1}\frac{f_{c}^{\prime }}{f_{fu}}\frac{E_{f}\varepsilon_{cu}}{E_{f}\varepsilon_{cu}+f_{fu}},$$

where *f_c_^′^* is specified compressive strength of concrete; *f_fu_* is the design tensile strength of FRP; *E_f_* is Young’s modulus of FRP bars, psi (MPa); *ε_cu_* is the ultimate compressive strain of concrete; *β_1_* is the ratio of the height of the equivalent rectangular stress block to the height of the neutral axis, which can be obtained from Equation (5):

(5) $$\beta_{1}=0.85-0.05\times (\frac{f_{c}^{\prime }-28}{7})\geq 0.65,$$

**Figure 5:**
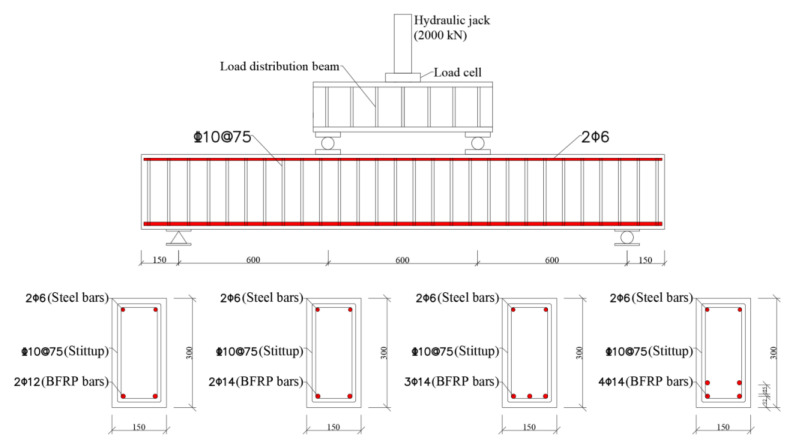
Dimensions and reinforcement details (all dimensions in millimeters).

All BFRP-SFRC beams were designed to flexure failure by concrete crushing. However, BFRP bars, concrete and construction quality were highly discrete; the actual design reinforcement ratio was greater than 1.4 times the balanced reinforcement ratio (*ρ_fb_).* Thus, the design balanced reinforcement ratio of the beam members was calculated by Equation (4). The design BFRP reinforcement ratios of the beams were 0.56%, 0.77%, 1.15% and 1.65%, respectively. Meanwhile, all beam specimens were reinforced with 10 mm diameter steel stirrups and had a spacing of 75 mm on the clear span to avoid shear damage. In addition, two steel bars of 6 mm diameter were placed on the top of the compression concrete region. The concrete cover was 15 mm. Figure 5 presents the dimensions and reinforcement details of all beam specimens. Table 4 shows the technical details of 11 beam specimens prepared for flexural testing as well as the actual concrete mechanical properties.

### 2.3. Test Setup and Instrumentation

**Figure 6:**
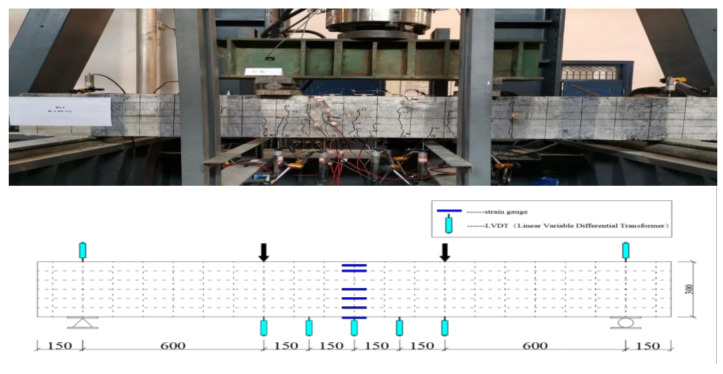
Measurement point setup (dimensions in millimeters).

Figure 6 shows the loading device and measurement point setup. All beams were simply supported on the steel frame. The 2000-kN Multifunctional Hydraulic Testing Machine (MHTM) at Zhengzhou University was used to load the midpoint of the steel distribution beams. Applied loads were transferred to the tested beams via a roller support and a hinge support; the two supports were spaced 600 mm. In order to monitor the concrete strain change of the mid-span section of the beams, six electrical strain gauges were attached along the beam’s height direction. The deflection of the beams was captured using seven linear variable differential transducers (LVDTs). The arrangement of LVDTs and strain gauges is shown in Figure 6. The specific values of load sensor, LVDTs and strain gauges were recorded by 3816 N Static Acquisition System of the Donghua Test Technology Company.

**Figure 7:**
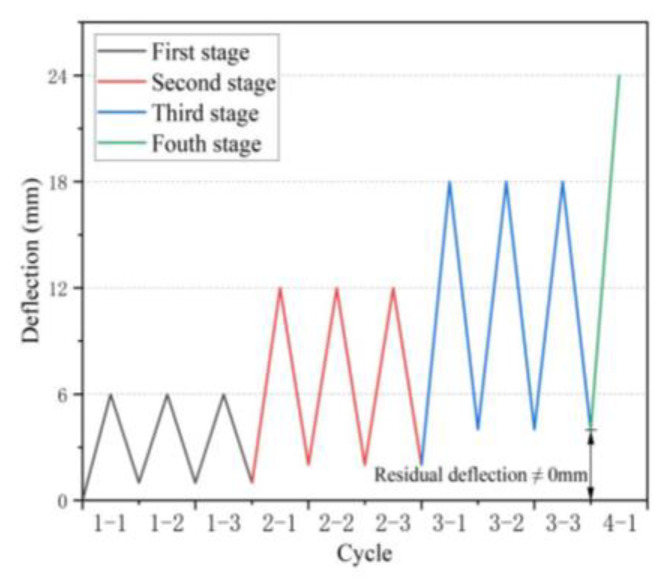
Loading and unloading system.

Before the formal test, except for three pre-cracked test beams, there were no cracks in the other test beams. All beams were loaded via four-point bending tests under a cyclic loading system. Figure 7 depicts the cyclic loading system. Initially, the load was applied using the load-controlled method (LCM) at a rate of 5 kN/min until the first crack appeared in the beam specimens. Soon afterward, the loading method was changed to displacement-controlled mode (DCM) at the speed of 2 mm/min until the mid-span deflection of the members reached 6 mm. Subsequently, the hydraulic actuator was unloaded to 0 kN at a pace of 5 mm/min. As mentioned above, three loading and unloading cycles were a complete stage, such a cyclic loading regime was imposed at the mid-span LVDT deflection increment of 6 mm in each stage until the beam lost carry capacity.

## 3. Results and Discussion

**Figure 8:**
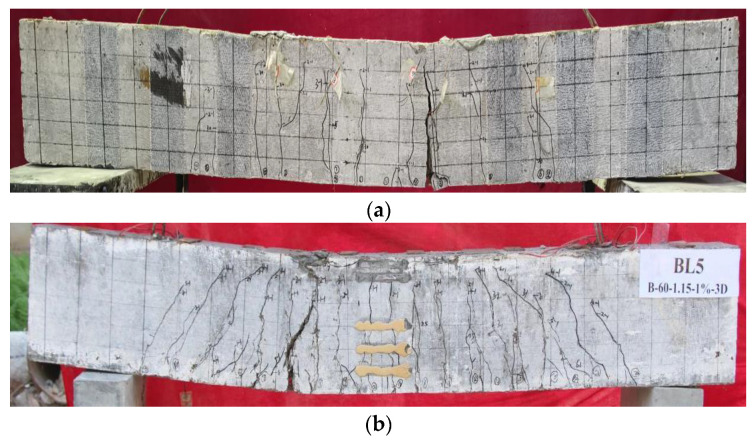
Failure modes of tested beams: (**a**) BFRP bars rupturing; (**b**) concrete crushing.

This section provides the experimental results of the 11 BFRP-SFRC beams in terms of cracking moment, ultimate moment capacity, load–deflection curves, envelope curves and residual deflections. Table 5 lists the failure modes, cracking moment, ultimate moment and deflections corresponding to the ultimate moment. The failure modes include BFRP bars rupturing and concrete crushing, as shown in Figure 8.

### 3.1. Cracking Moment

**Figure 9:**
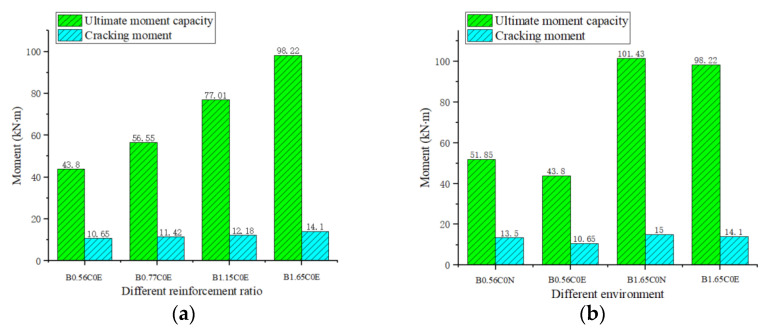
Cracking moment and ultimate moment capacity of specimens: (**a**) BFRP reinforcement ratio; (**b**) Environment.

For the RC structures of seawater erosion, once cracks occur, the structures will be irreversibly damaged. So, the cracking moment of BFRP-SFRC beams in the marine environment was an important index. The cracking moment (M_cr_)was defined as when the BFRP bars strain increased suddenly or the first crack appeared in the bottom concrete. It can be seen from Figure 9a that the BFRP reinforcement ratios had little influence on the cracking moment of the specimens. The reason is that the cracking moment was only related to the tensile strength of concrete, while the BFRP reinforcement ratios had no effect on the tensile strength of concrete. From Figure 9b, there was no significant difference in the cracking moment of the test beams in different environments. This is because steel fiber-reinforced concrete with a low water binder ratio in this paper had low porosity, which made it impossible for chloride ions to penetrate the concrete. When the tested specimens were immersed in simulated seawater solution, only the 2–3 mm thick concrete on the surface was eroded [23]. Therefore, chloride ions in the marine environment had little influence on the mechanical properties of steel fiber-reinforced concrete, which meant that the marine environment had little influence on the cracking moment.

### 3.2. Load-Deflection Curves

**Figure 10:**
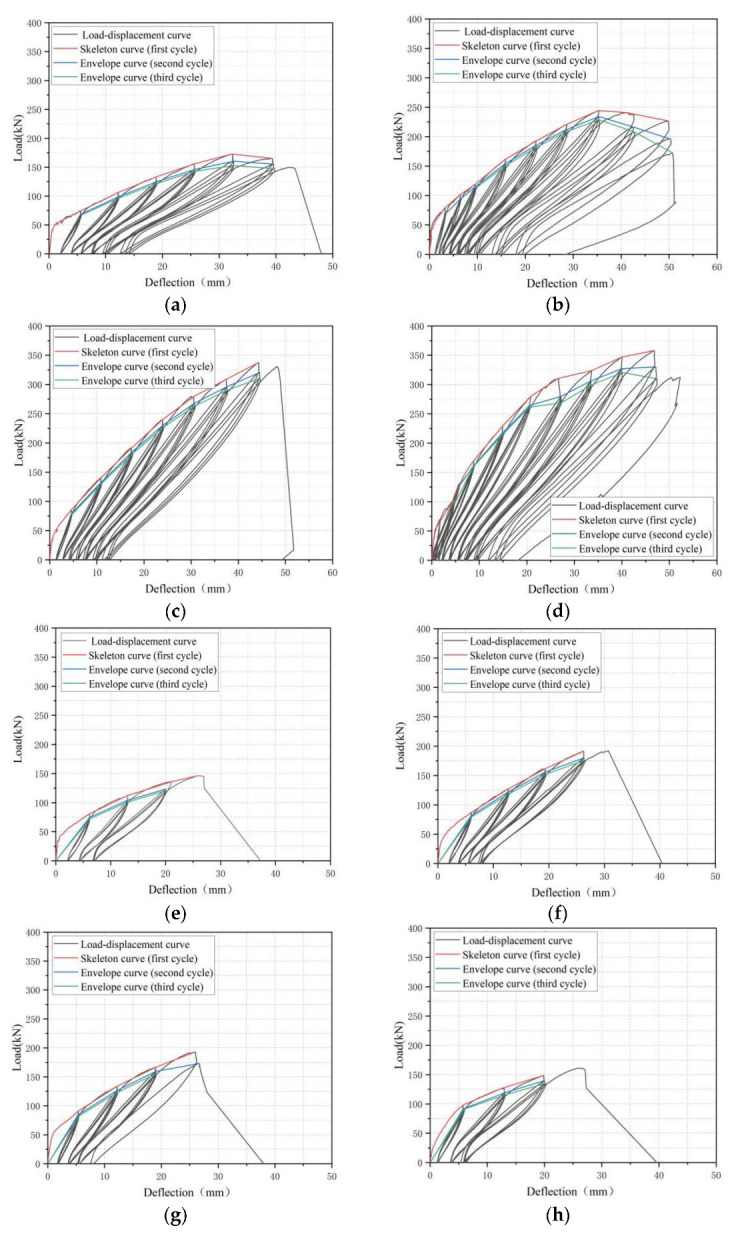

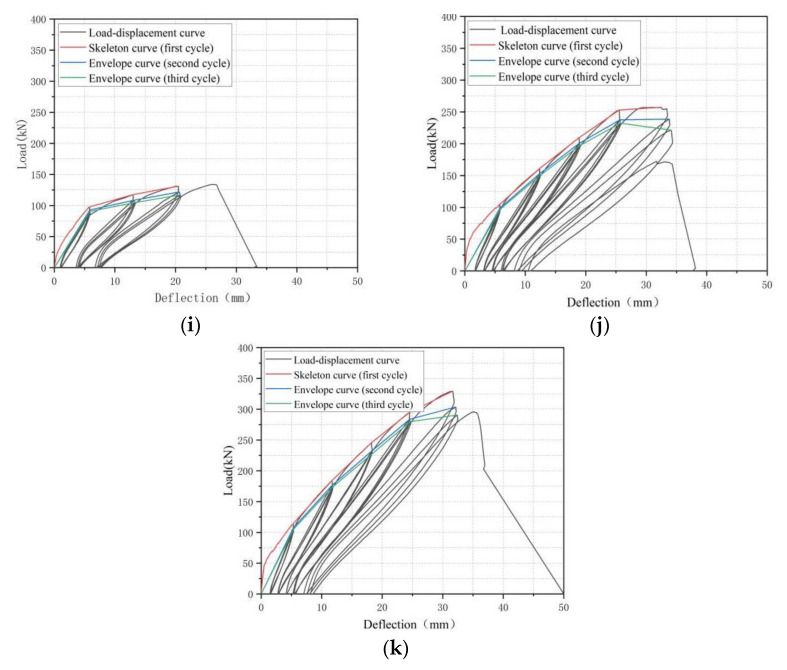
Load–deflection curves for all beam specimens: (**a**) B0.56C0N; (**b**) B0.77C0N; (**c**) B1.15C0N; (**d**) B1.65C0N; (**e**) B0.56C0E; (**f**) B0.77C0E; (**g**) B0.77C0.02E; (**h**) B0.77C0.2E; (**i**) B0.77C0.4E; (**j**) B1.15C0E; (**k**) B1.65C0E.

The load–deflection curves (half hysteretic curve) of BFRP-SFRC beams under cyclic loading was an important way to analyze the envelope curves, residual deflection, stiffness, etc. The load–deflection curves of all beams are shown in Figure 10. The envelope curves were defined as the curve in which the peak loads were connected successively for each cycle in the load–deflection curves. The red, blue and green curves represent the first cyclic envelope, the second cyclic envelope and the third cyclic envelope of the load–deflection curves of all tested specimens, respectively. Residual deflection was defined as the irrecoverable plastic deflection of the beam when the load unloads to 0 kN.

From Figure 10, it can be clearly seen that the load–deflection curves of all beams under cyclic loading had the same characteristics. Firstly, the peak load decreased with the increase in loading and unloading cycles under the same displacement. At the same time, with the increase in deflection, the impact of the loading and unloading cycles on the peak load gradually increased. This was mainly due to the rapid increase in residual deflection and the residual crack of the beams after the first loading and unloading, which led to the stiffness of the test beams gradually decreasing, and the peak load value decreasing with the same deflection. In addition, with the increase in deflection, the neutral axis of the test beam section moved up, and the stiffness decreased, which made the influences of the loading and unloading cycles on the peak load under the same deflection increase. Secondly, with the increase in deflection, the influence of loading and unloading cycles on the residual deflection of the test beam increased. For example, the residual deflection of tested beam B1.15C0E under the third loading and unloading was 2.1% higher than that under the first loading and unloading at the 6 mm mid-span deflection load level, while it was 7.9% greater at the 32 mm mid-span deflection load level. This was due to the increase in deflection, resulting in a rapid reduction in the stiffness of the test beams, and the residual deflection increased after increasing the loading and unloading cycles. Thirdly, as the deflection of beams increased, the area covered by load–deflection curves increased and the energy consumption increased. The reason was that the increasing deflection made the peak load and deformation of the test beams increase, and the area included in the load displacement curves increased, which led to the energy consumption increasing. Finally, with the increase in numbers of loading and unloading cycles under the same deflection, the energy consumption of the test beam decreased. With the increase in numbers of loading and unloading cycles, the peak load of beams decreased, but the residual deflection increased, which reduces the area enclosed by the load–deflection curves and energy consumption. For instance, the area enclosed by the first loading–unloading load–deflection curve of tested beam B1.15C0E was 2% higher than that of the third at 6 mm mid-span deflection load level.

### 3.3. Skeleton Curves

**Figure 11:**
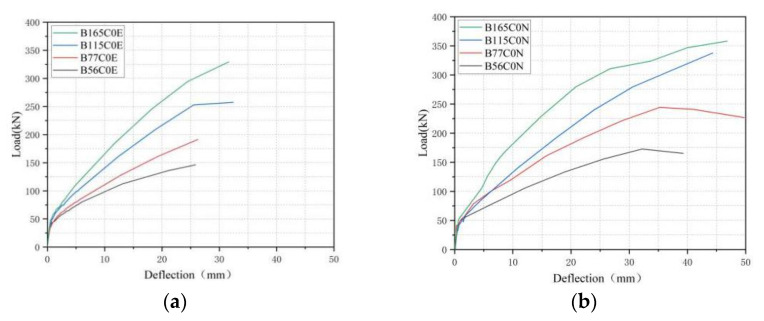

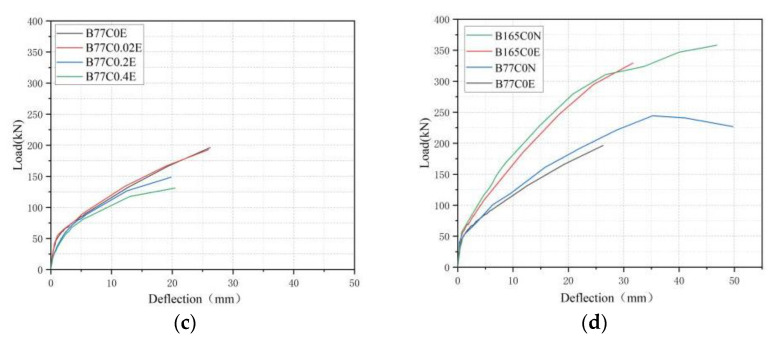
Skeleton curve for beams with different parameters: (**a**) BFRP reinforcement ratio (erosion environment); (**b**) BFRP reinforcement ratio (natural environment); (**c**) Pre-cracked widths and (**d**) Environment.

The first cycle envelope was defined as the skeleton curves. Figure 11 describes the skeleton curves of all the specimens. From Figure 11, the skeleton curves of the tested beams had a similar trend, showing a linear growth trend after cracking. The peak load with the same deflection decreased significantly with the increase in the number of loading and unloading cycles.

From Figure 11a,b, it can be found that, as the BFRP reinforcement ratios increased, the slope of curves was larger, which indicated that increasing the BFRP reinforcement ratios can effectively enhance the flexure stiffness of the specimens after cracking. Therefore, the specimens with high reinforcement ratios had a better ability to resist deformation. Meanwhile, a certain bond-slip and friction between the BFRP bars and the concrete constantly occurred in the loading process, which resulted in the concrete cracking in the tension zone, so that the flexure stiffness of the specimen decreased with the increase in the load level. The deflection of beams B0.77C0E, B1.15C0E and B1.65C0E were 19.6%, 47.9% and 58.9% lower than that of B0.56C0E at the load of 100 kN, respectively.

The skeleton curves of the tested specimens with different pre-cracked widths are displayed in Figure 11c. It can be seen that the slope of the skeleton curves for the beams with pre-cracked widths of 0 mm and 0.02 mm were greater than that of the beams with pre-cracks of 0.2 mm and 0.4 mm. With the increase in the load level, the stiffness of the tested beams with a larger pre-crack width degraded rapidly. This was because the initial stiffness of the specimens was different due to the pre-splitting treatment, and the initial stiffness decreased with increasing pre-cracked widths. The simulated seawater solution entered the tested beams through the pre-cracked widths and caused a decrease in the tensile strength and elastic modulus of the BFRP bars. The larger the pre-cracked widths, the more serious the sea water erosion to the tested beam. The deflection of beams B0.77C0.4E were 34.0% lower than that of B0.77C0E at the load of 100 kN.

The skeleton curves of the tested beams in different environments are shown in Figure 11d. It can be seen that the simulated seawater erosion had no significant impact on the stiffness before concrete cracking. This was because the initial stiffness of the tested beams was only related to the BFRP reinforcement ratios and elastic modulus of the concrete, and seawater erosion had no significant impact on the elastic modulus of the concrete. However, the stiffness of the tested beams corroded by seawater after cracking was less than that of the tested beams not corroded by seawater. This was because the stiffness after cracking was related to the crack development speed. The crack development speed of the tested beams corroded by seawater was greater than that of the tested beams not corroded by seawater. The deflection of beams B0.77C0E were 13.1% higher than that of B0.77C0N at the load of 100 kN.

### 3.4. Residual Deflection

**Figure 12:**
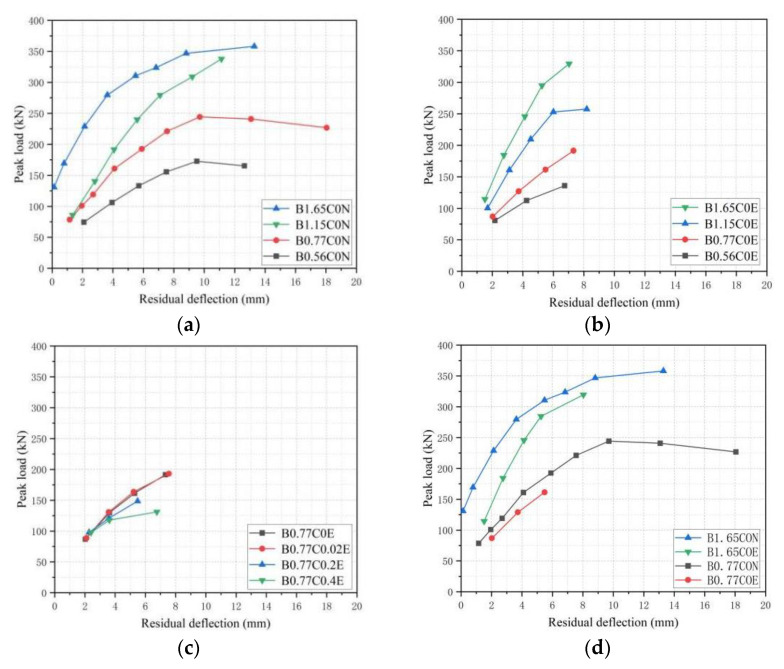
Peak load–residual deflection curves (first cycle) for beams with different: (**a**) BFRP reinforcement ratio (erosion environment); (**b**) BFRP reinforcement ratio (natural environment); (**c**) Pre-cracked widths; and (**d**) Environment.

Figure 12a,b shows the peak load–residual deflection curves of different BFRP reinforcement ratios. For BFRP-SFRC beams with different reinforcement ratios (B0.56C0N, B0.77C0N, B1.15C0N, B1.65C0N and B0.56C0E, B0.77C0E, B1.15C0E, B1.65C0E), the residual deflection had an obvious relationship with the BFRP reinforcement ratios. The specimens with a higher reinforcement ratio produced smaller mid-span displacement and less residual deflection after unloading under the same loading level, Therefore, the specimen with a higher reinforcement ratio has better resistance to deformation. Compared with different loading levels, the residual deflection of the tested beam specimens with high reinforcement ratios increased slowly, and to generate the same residual deflection, the specimens with a high reinforcement ratio needed to apply a larger load. This reason was that the strain growth rates of the tested beams with lower BFRP reinforcement ratios were greater than that of the tested beams with a high-reinforcement ratio. The strain of the tested beams with lower BFRP reinforcement ratios were larger and the BFRP bars produced irreversible damage under the same load, which resulted in a larger residual deflection after loading and unloading cycles. The residual deflection of B1.65C0E, B1.15C0E and B0.77C0E under the 100 kN load was 61.5%, 51.2% and 25.2%, respectively, lower than that of B0.56C0E.

Figure 12c illustrates peak load–residual deflection curves for different pre-cracked widths. Due to the bearing capacity of the tested beams and the stress of the BFRP reinforcement being low at the 6 mm and 12 mm displacement loading levels, the effect of the pre-crack width of the specimens on the residual deflection was not obvious. Although basalt fiber was a stable amorphous material, the tested beam specimens with larger pre-cracked widths also had more initial cracks, so the erosion of BFRP bars by simulated seawater was more serious, resulting in their tensile strength and Young’s modulus decreasing. The plastic deformation and residual deflection of the experimental beams with a large pre-crack width were significantly larger than those with a small pre-crack width under the same load level. The residual deflections of the tested beam B0.77C0E were 1.3%, 5.17% and 29.6% lower than that of the tested beams B0.77C0.02E, B0.77C0.2E and B0.77C0.4E at the 20 mm displacement loading level, respectively.

Figure 12d presents the peak load–residual deflection curves of the tested beam specimens in different environments. From Figure 12d, it can be seen that the residual deflection of the simulated seawater erosion-tested beams were larger than that of the natural state-tested beams.

The stiffness degradation of the specimens immersed in the simulated seawater for one year was faster than that of the tested beams in the natural state, and the resulting deflection and the residual deflection after unloading were larger. The residual deflection of the tested beam B1.65C0E was 386.6% larger than that of the tested beam B1.65C0N at a load of 150 kN. The residual deflection of the tested beam B0.77C0E was 38.2% larger than that of the tested beam B0.77C0N at a load of 100 kN.

### 3.5. Energy Consumption Capacity

**Figure 13:**
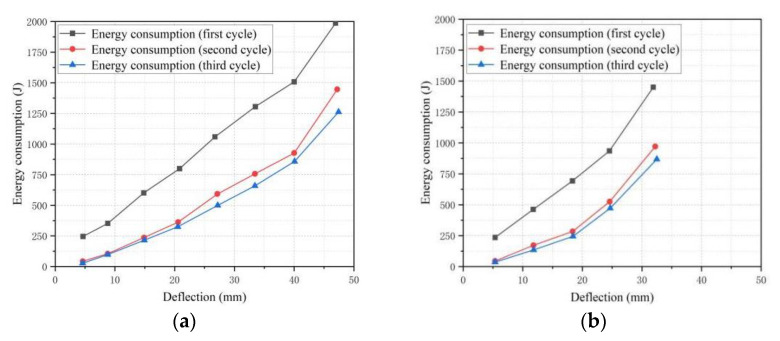
Energy consumption—deflection curves for beams: (**a**) B1.65C0N; (**b**) B1.65C0E.

Energy consumption was also an important index of the flexural performances of the beams. The hysteretic decay of the BFRP-SFRC beams occurred during the loading and unloading cycle, manifested as the reduction in the area of the hysteretic loop, the attenuation of the bearing capacity and the degradation of the stiffness. Generally, the decrease in hysteresis capacity of the BFRP-SFRC beams is regarded as its damage development process, so the loss of energy consumption capacity could be used as the damage index to evaluate the damage degree of the tested beam specimens. The area enclosed by the load–deflection curves (hysteresis curve) was the energy consumed by the tested beams in this cycle after the loading and unloading cycle was completed. According to the maximum mid-span displacement in each cycle, draw the energy consumption-maximum displacement diagram of partial specimen, as shown in Figure 13, From Figure 13, it can be seen that the energy consumption capacity of the tested beams increased with the increase in the displacement; especially when the mid-span deflection was greater than 25 mm, when the energy consumption increased exceptionally rapidly. The reason was that when the load was less than 30% of the ultimate load, the crack width developed slowly and the plastic energy consumed by the crack was less. With the continuous increase in the load, the stiffness of the tested beams decreased sharply, the crack developed sufficiently and the energy consumed by the crack increased significantly. All the cracks in the first cycle were newly developed cracks, and the cracks in the second cycle and the third cycle were a small part of the cracks developed based on the residual cracks, and the maximum load of the second cycle and the third cycle are less than the first cycle because of the loading regime in this study. So, the energy consumption capacity of the first cycle was significantly greater than that of the second and third cycle.

**Figure 14:**
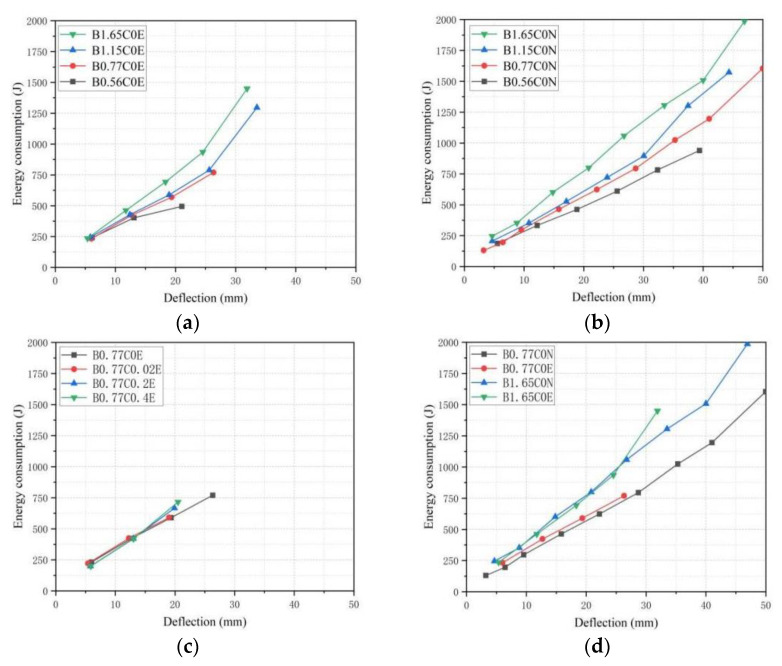
Energy consumption—deflection curves (first cycle) for beams with different: (**a**) BFRP reinforcement ratio (erosion environment); (**b**) BFRP reinforcement ratio (natural environment); (**c**) Pre-cracked widths; and (**d**) Environment.

Figure 14a,b shows the energy consumption–deflection curves of different BFRP reinforcement ratios. For the BFRP-SFRC beams with different reinforcement ratios (B0.56C0N, B0.77C0N, B1.15C0N, and B1.65C0N; B0.56C0E, B0.77C0E, B1.15C0E and B1.65C0E), their energy consumption capacity had an apparent relationship with the reinforcement ratio of BFRP bars. The flexural stiffness of the specimens with a high reinforcement ratio was large. Compared with the tested beam specimens with a low reinforcement ratio, the tested beams with high BFRP reinforcement ratios needed more load to achieve the same displacement, so the energy consumption capacity of the beams was positively related to the BFRP reinforcement ratios. The reason was that the total strain energy stored in the BFRP bars and concrete of the tested beam specimens with low-reinforcement ratios were smaller than that of the tested beam specimens with high-reinforcement ratios during loading. Under different displacement-loading levels, with the BFRP reinforcement ratios increasing, the rate of increment of energy consumption became bigger, and under the condition of the same energy consumption, the specimens with a higher BFRP reinforcement ratio required less deflection. The deflections of the tested beams B1.65C0E, B1.15C0E and B0.77C0E were 39.3%, 27.3% and 23.3% lower than those of the tested beam B0.56C0E.

The energy consumption–deflection curves for different pre-cracked widths are shown in Figure 14c. From Figure 14, it can be seen that before the 12 mm displacement loading level, due to the lower bearing capacity of the tested beams and the lower strain of the concrete and BFRP bars, the pre-cracked widths had no pronounced effect on the energy consumption of the structure. With the displacement loading level increasing, the BFRP bars produced a large tensile deformation. The energy consumption of the tested beams with a large pre-crack width was slightly lower than that of the tested beams with a small pre-crack width under the same displacement, because the tested beams with a large pre-crack width seriously eroded the simulated seawater solution. Significantly, the tensile strength and Young’s modulus of the BFRP bars were obviously degraded. As a result, the ultimate flexure capacity of the tested beams was also decreased accordingly, ultimately manifested in the weakened energy consumption capacity of the beams. The energy consumed by the tested beam B0.77C0E was 1.5%, 7.8% and 11.0% higher than that of the tested beams B0.77C0.02E, B0.77C0.2E and B0.77C0.4E at the 18 mm displacement loading level, respectively.

Figure 14d displays the energy consumption–deflection curves of the tested beams in different environments. For the tested beams with the same reinforcement ratios, the seawater erosion had no marked effect on the energy consumption capacity of the tested beams at the same deflection. However, the tested beams in seawater erosion had a lower ultimate flexure capacity, which led to a reduction in the number of loading and unloading cycles, which significantly reduced the total energy consumption capacity. The total energy consumption of B0.77C0E and B1.65C0E was 52% and 27% lower than that of B0.77C0N and B1.65C0N, respectively. It can be found that the effect of sea water erosion on energy consumption of tested beams with high BFRP reinforcement ratios were smaller than that of tested beams with low BFRP reinforcement ratios.

## 4. Comparison of Experimental Results with Model Predictions

As we all know, the stress and strain of FRP bars increase linearly, which makes the load–displacement curves of FRP-RC beams different from that of RC beams, showing a bilinear growth. Meanwhile, the Young’s modulus of the FRP bars is lower than that of steel bars, which makes the deformation of FRP-RC beams under the same load greater than that of RC beams with the same reinforcement ratio, but the ultimate bearing capacity was higher than that of RC beams. Therefore, the design of FRP-RC beams is usually controlled by the serviceability limit states, and deformation is a critical control parameter in the design of FRP-RC members. Nowadays, there are four methods to calculate the deflection of the FRP-RC beams, including effective moment of inertia method, stiffness analysis method, bilinear method and curvature integral method. Most of the literature and the codes used the effective moment of inertia method to calculate the deflection of FRP-RC beams under transient and continuous static loads in the serviceability limit states. This paper used the effective moment of inertia method to evaluate the serviceability deflection of BFRP-SFRC beams under the coupled action of cyclic loading and simulated seawater erosion. The results of various model predictions and new evaluation methods were compared with the experimental results of this study.

### 4.1. Theoretical Calculation Models of FRP-RC Beam Deflection

According to the linear elastic analysis of material mechanics, the mid-span deflection of FRP-RC beams under four-point loading test can be obtained by Equation (6) [28]:

(6) $$\Delta =\frac{FS}{48E_{c}I_{e}}(3L^{2}-4S^{2}),$$

where *F* is the applied load; *S* is the length of the beam shear span; *L* is the calculated length of the beam; *E_c_* is the elastic modulus of concrete; and *I_e_* is the effective moment of inertia.

The methods recommended by ACI 440.1R-15 [27], ACI 440.1R-03 [29] and H.K. Ammash et al. [30] were summarized and investigated.

The initial formula *I_e_* was proposed by Branson [31], which is expressed as the weighted average of the stiffness of cracked and cracked members. ACI 440.1R-03 [29] introduces the reinforcement ratio correction factor *β_d_* when calculating the *I_e_* of FRP-RC beam. ACI 440.1R-03 [29] recommended that Equation (7) should be used to calculate *I_e_*:

(7) $$I_{e}={\left(\frac{M_{cr}}{M_{a}}\right)}^{3}\beta_{d}I_{g}+\left[1-{\left(\frac{M_{cr}}{M_{a}}\right)}^{3}\right]I_{cr}\leq I_{g},$$

(8) $$\beta_{d}=\alpha_{b}\left[\frac{E_{f}}{E_{s}}+1\right]\leq 1,$$

where $\alpha_{b}=0.5$; *M_cr_* is the cracking moment; *M_a_* is the applied moment; *I_g_* is the moment of inertia of gross cross-sectionl *I_cr_* is the moment of inertia of cracked cross-section, determined as:

(9) $$I_{cr}=\frac{b}{3}d^{3}k^{3}+n_{f}A_{f}d^{2}{\left(1-k\right)}^{2},$$

(10) $$k=\sqrt{2\rho_{f}n_{f}+{\left(\rho_{f}n_{f}\right)}^{2}}-\rho_{f}n_{f},$$

(11) $$n_{f}=\frac{E_{f}}{E_{c}},$$

where *d* is the distance from extreme compression fiber to centroid of tension reinforcement; *k* is the ratio of the depth of neutral axis to reinforcement depth; *n_f_* is the ratio of modulus of elasticity of FRP bars to the modulus of elasticity of concrete; *A_f_* area of fiber-reinforced polymer (FRP) reinforcement; *ρ_f_* is the fiber-reinforced polymer reinforcement ratio; *E_f_* is design or guaranteed modulus of elasticity of FRP defined as mean modulus of the sample of test specimens; *E_c_* is the modulus of elasticity of concrete.

ACI 440.1R-03 [29] recommends that the cross-section be considered homogeneous before the member concrete cracks, and the effect of FRP bars on the total moment of inertia of the section should be ignored. Therefore, the total moment of inertia of the cross-section can be calculated by the following formula:

(12) $$I_{g}=\frac{bh^{3}}{12},$$

where *b* is the width of the beam and *h* is the total height of the beam.

The cracking moment *M_cr_* is as specified in ACI 318-19 [32] and should be computed using Equation (13):

(13) $$M_{cr}=\frac{0.62\lambda \sqrt{f_{c}^{\prime }}I_{g}}{y_{t}},$$

where *λ* is the modification factor reflecting the reduced mechanical properties of lightweight concrete; *f_c_^′^* is specified compressive strength of concrete; *y_t_* is the distance from the centroidal axis of the gross section, neglecting reinforcement, to the tension face.

ACI 440.1R-15 [27] gives another *I_e_* calculation equation, Equation (14), for the FRP-RC beam, and introduces a coefficient *γ* to consider the variation in stiffness along the length of the specimen:

(14) $$I_{e}=\frac{I_{cr}}{1-\gamma {\left(\frac{M_{cr}}{M_{a}}\right)}^{2}\left[1-\frac{I_{cr}}{I_{g}}\right]}\leq I_{g},$$

where *γ* is a parameter dependent on load and boundary conditions and accounts for the length of the uncracked regions of the member and for the change in stiffness in the cracked regions, determined as:

(15) $$\gamma =1.72-0.72\left(\frac{M_{cr}}{M_{a}}\right),$$

H.K. Ammash et al. [30] proposed that the effective moment of inertia (*I_e,Ammash_*) of an FRP-RC beam section can be evaluated by Equation (16):

(16) $$I_{e{,}_{Ammash}{}_{.}}={\left(\frac{M_{cr}}{M_{a}}\right)}^{\alpha }I_{g}\chi +\left(\chi -{\left(\frac{M_{cr}}{M_{a}}\right)}^{\alpha }\right)I_{cr}\leq I_{g},$$

(17) $$\alpha =5-0.03\lambda f_{c}^{\prime }-\frac{h}{L};\chi =1-\frac{h}{L},$$

(18) $$\lambda =1.0\mathrm{for}\;f_{c}^{\prime }\leq 40Mpa,$$

(19) $$\lambda =0.8\mathrm{for}\;f_{c}^{\prime }\geq 40Mpa,$$

where *α* is a coefficient related to beam height, span and concrete strength; *χ* is a coefficient related to the depth and span of the beam; *λ* is a factor associate with concrete strength.

H.K. Ammash et al. [30] used Equation (20) to calculate the deflection of the FRP-RC beam:

(20) $$\Delta =\frac{CM_{a}L^{2}}{E_{c}I_{e}}\zeta \psi ,$$

where *C* is a constant for moment calculation depend on loading type, which: *C* = 5/48 for uniform load, *C* = 1/12 for three-point loading. *C* = 23/216 for four-point loading; *ζ* is a factor adapt for the effect of loading type, which: *ζ* = 0.9 for uniform load, *ζ* = 1.15 for three-point loading, *ζ* = 1.0 for four-point loading; *ψ* is a coefficient accommodate the effect of compressive strength and modulus of elasticity, where: *ψ*= 1.0 for normal concrete, *ψ*= 1.25 for high-strength concrete.

The tensile strength of plain concrete after cracking was generally neglected owing to its low tensile strength when calculating the deflection of the FRP-RC beams. However, the steel fiber was added to the concrete matrix, which induced the strain-hardened characteristics of concrete. The contribution of the tensile strength of SFRC to the flexural resistance of FRP-RC beams cannot be ignored. Therefore, the equations proposed by the above specifications and scholars was no longer applicable to predict the deflection of BFRP-SFRC beams under the coupling effect of cyclic loading and simulated seawater erosion.

### 4.2. New Calculation Method of Deflection

Due to the tensile stress that can be carried by the steel fibers in the fractured section after the concrete had cracked, the contribution of the steel fibers to the tensile region should be considered when calculating the deformation. However, the position and orientation of the steel fibers in the concrete matrix were random, which made it impossible to calculate the area and moment of inertia of a single steel fiber. When calculating the deformation of the beam, the steel fiber can usually be taken as a whole [33]. In addition, the contribution of steel fibers to concrete in the tension zone mainly depended on two factors, including distribution and orientation of the steel fibers. The distribution of steel fibers is usually represented by the non-uniformity coefficient *η_v_*, and the orientation of the steel fibers can be represented by the orientation coefficient *η_0_*. Gao [34] suggested using Equation (21) to calculate the total area of steel fibers in the beam cross-section:

(21) $$A_{sf}=\eta_{0}\eta_{v}bhV_{sf}=\eta bhV_{sf},$$

where *η* defined as the effective coefficient of steel fibers; *V_sf_* is the actual volume fraction of steel fibers in the cast concrete.

Since the steel fiber area of the whole cross-section of BFRP-SFRC beams was reduced after the pre-splitting treatment, Equation (22) was modified according to the experimental results. The modified total area of steel fibers can be calculated by the following formula:

(22) $$A_{sf}=(1-\omega_{pre-split\;width})\eta_{0}\eta_{v}bhV_{sf}=(1-\omega_{pre-split\;width})\eta bhV_{sf},$$

Zhang [35] suggested that for hooked steel fibers, the effective coefficient of steel fibers was between 0.16–0.33. The effective coefficient of steel fibers *η* in this study was taken as 0.17.

**Figure 15:**
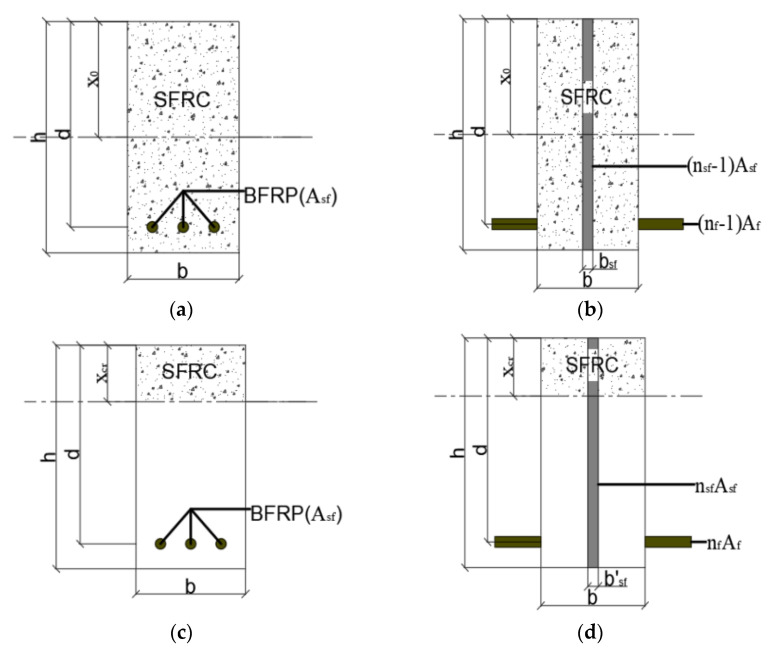
Calculations for uncracked and cracked sections: (**a**) Gross section; (**b**) Transformed uncracked section; (**c**) Cracked section; (**d**) Transformed cracked section.

The gross section and the converted equivalent section of the BFRP-SFRC beams are shown in Figure 15a,b. According to the condition that the area moments of the tension zone and the compression zone on the cross-section were equal, Equation (23) can be deduced:

(23) $$\frac{1}{2}bx_{0}^{2}+\frac{1}{2}b_{sf}x_{0}{}^{2}=\frac{1}{2}b{\left(h-x_{0}\right)}^{2}+(n_{f}-1)A_{f}(d-x_{0})+\frac{1}{2}b_{sf}{\left(h-x_{0}\right)}^{2},$$

The height of the compression zone before cracked can be deduced from Equation (24):

(24) $$\left\{\begin{array}{l} x_{0}=\frac{\frac{1}{2}bh^{2}+(n_{f}-1)A_{f}d+\frac{1}{2}b_{sf}h^{2}}{bh+(n_{f}-1)A_{f}+b_{sf}h}\\ b_{sf}=\frac{(n_{sf}-1)A_{sf}}{h} \end{array}\right.,$$

where *x*_0_ is the depth of the compression zone of the total section before being cracked; *A_sf_* is the area of all steel fibers in the cross-section; *b_sf_* is the width of steel fiber of the uncracked section after being transformed; *n_sf_* is the modulus ratio between the steel fiber *E_f_* and the steel fiber reinforced concrete *E_c_*.

The moment of inertia of BFRP-SFRC beams can be calculated by Equation (25):

(25) $$I_{g}=\frac{b}{3}\left[x_{0}^{3}+{\left(h-x_{0}\right)}^{3}\right]+(n_{f}-1)A_{f}{\left(d-x_{0}\right)}^{2}+\frac{b_{sf}}{3}\left[x_{0}{}^{3}+{\left(h-x_{0}\right)}^{3}\right],$$

The cracked section and converted equivalent section of the BFRP-SFRC beams are shown in Figure 15c,d. According to the condition that the area moments of the tension zone and the compression zone on the cross-section were equal, Equation (26) can be deduced:

(26) $$\frac{bx_{cr}^{2}}{2}+\frac{b_{sf}x_{cr}^{2}}{2}=n_{f}A_{f}(d-x_{cr})+\frac{b_{sf}^{\prime }{\left(h-x_{cr}\right)}^{2}}{2},$$

(27) $$b_{sf}^{\prime }=\frac{n_{sf}A_{sf}}{h},$$

where *b_sf_^′^* is the width of steel fiber of the racked section after being transformed.

The height of the compression zone after cracking can be deduced by Equation (28):

(28) $$x_{cr}=\frac{-(n_{f}A_{f}+b_{sf}^{\prime }h)+\sqrt{{\left(n_{f}A_{f}+b_{sf}^{\prime }h\right)}^{2}+2(b+b_{sf}-b_{sf}^{\prime })(n_{f}A_{f}d+\frac{b_{sf}^{\prime }}{2}h^{2})}}{b+b_{sf}-b_{sf}^{\prime }},$$

where *x_cr_* is the depth of compression zone after being cracked.

The moment of inertia of cracked section of BFRP-SFRC beams can be calculated by Equation (29):

(29) $$I_{cr}=\frac{b}{3}x_{cr}^{3}+\frac{b_{sf}}{3}x_{cr}^{3}+n_{f}A_{f}{\left(d-x_{cr}\right)}^{2}+\frac{b_{sf}^{\prime }}{3}{\left(h-x_{cr}\right)}^{3},$$

Toutanji et al. [36] considered that the effective moment of inertia of FRP-RC beams can be calculated by the following equation:

(30) $$I_{e,\mathrm{Toutanji}}={\left(\frac{M_{cr}}{M_{a}}\right)}^{m}I_{g}+\left[1-{\left(\frac{M_{cr}}{M_{a}}\right)}^{m}\right]I_{cr}\leq I_{g},$$

where $m=6-10\frac{E_{FRP}}{E_{s}}\rho_{FRP}\;\mathrm{if}\;\frac{E_{FRP}}{E_{s}}\rho_{FRP}≻0.3$ Or *m* = 3.

The deflection of the BFRP-SFRC beams with different pre-cracked widths, environment and BFRP reinforcement ratios can be calculated by Equations (6), (25), (29) and (30). Table 6 summarizes the various models from the design codes and the literature, as well as the new calculation method proposed in this study, which calculates the deflection of all tested beams when the stroke of the actuator reached 6 mm. As can be seen from Table 6, the calculated deflections of ACI 440.1R-15 [27], ACI 440.1R-03 [29] and H.K. Ammash et al. [30] were 11–32% higher than the experimental values because the influence of the steel fibers was not considered. The mid-span deflections calculated by the new method proposed in this paper agree well with the experimental results, whether it was simulated seawater erosion or natural environment.

## 5. Conclusions

This paper experimentally explored the effects of BFRP reinforcement ratios, pre-cracked widths and simulated seawater erosion on the flexure behaviors of BFRP-SFRC beams. A total of eleven BFRP-SFRC beams were tested via four-point bending under cyclic loading, including four beams with the natural environment and seven beams corroded by simulated seawater solution. Meanwhile, to accurately evaluate the serviceability deflection of BFRP-SFRC beams, a new deflection analysis model based on the effective moment of inertia method was proposed. Based on the experimental results and discussions presented in this research, the following conclusions and recommendations can be drawn:

1. The cracking load of BFRP-SFRC beams was independent of the BFRP reinforcement ratios, at the same time, the seawater erosion had little effect on the cracking load after one year. The cracking load was mainly related to the tensile strength of concrete;
2. The load–deflection curves of BFRP-SFRC beams show bilinear growth. With the increase in the loading and unloading cycles, the peak load and energy consumption of the tested beams decreased, while the residual deflection increased, and the impact of loading and unloading cycles on the flexural performances of the BFRP-SFRC beams enhanced with the increase of displacement;
3. High BFRP reinforcement ratios were conducive to increasing the stiffness and total energy consumption, reducing the deformation and the residual deflection of the test beams. The peak load and energy consumption of BFRP-SFRC beams after seawater erosion decreased, but the stiffness change was not obvious under the same displacement;
4. The contribution of steel fibers to the stiffness of the beams was ignored at the after cracking when using the current effective moment of inertia model to calculate the moment of inertia of the BFRP-SFRC beams. Considering the pre-splitting treatment, seawater erosion and the strain-hardening behavior of steel fibers after cracking, a new deflection evaluation model of BFRP-SFRC beams was established, whose results were closer to the experimental values than those of other available models.

In further studies, the authors intend to investigate the influence of pre-cracked width on the flexural behaviors of ultra-high-performance concrete beams reinforced with FRP bars subjected to seawater and alkaline environments. Moreover, the effects of porosity of section of beams and the times of repeated loading on flexural behaviors will be studied as well.

## Figures and Tables

**Table 1:** Concrete mix proportion of the specimens (kg/m^3^).

Water	Cement	Coarse Aggregate	Sand	Steel Fiber	Water-Reducers
164	529	1026	706	78.5	5.82

**Table 2:** Mechanical properties of the BFRP bars.

*d_f_* (mm)	*A_f_* (mm²)	Mechanical Properties of BFRP Bars			
		Before Seawater Erosion		After Seawater Erosion (365 d)	
		*f_fu_* (MPa)	*E_f_* (GPa)	*f_fu_* (MPa)	*E_f_* (GPa)
12	113.1	1034.1	43.26	926.9	39.4
14	153.9	1025.6	41.79	910.8	38.2

**Table 3:** Components of Simulated Seawater.

Kind	Content (g/L)	Kind	Content (g/L)
NaCl	28.219	MgCl_2_	2.392
MgSO_4_	3.439	CaCl_2_	1.234
NaHCO_3_	0.256	KCl	0.764

**Table 4:** Technical details of all specimens.

Series	Specimens	BFRP Reinforcement Ratios (%)	Pre-Crack Width (mm)	Environment	Actual Foundation Mechanical Properties of Concrete			
					Before Seawater Erosion		After Seawater Erosion	
					*f_cu_* (MPa)	*f_c_* (MPa)	*f_cu_* (MPa)	*f_c_* (MPa)
I	B0.56C0N	0.56	0	Nature	48.13	60.16		
	B0.77C0N	0.77	0	Nature	52.45	74.99		
	B1.15C0N	1.15	0	Nature	65.18	81.47		
	B1.65C0N	1.65	0	Nature	61.18	76.47		
II	B0.56C0E	0.56	0	Seawater erosion	64.58	41.45	77.24	55.16
	B0.77C0E	0.77	0	Seawater erosion	74.99	54.45	87.50	65.62
	B0.77C0.02E	0.77	0.02	Seawater erosion	72.13	52.93	83.13	70.64
	B0.77C0.2E	0.77	0.2	Seawater erosion	66.98	50.69	81.69	65.71
	B0.77C0.4E	0.77	0.4	Seawater erosion	69.16	52.42	84.17	68.41
	B1.15C0E	1.15	0	Seawater erosion	69.31	53.84	83.37	63.12
	B1.65C0E	1.65	0	Seawater erosion	72.56	53.14	86.61	67.76
Average value					65.15	59.27	83.39	65.20
Standard deviations					8.39	12.67	3.38	5.03

**Table 5:** Experimental results of tested specimens.

Series	Specimens	Failure Mode	*M_cr_* (kN)	*M_u_* (kN·m)	Δ*_max_* (mm)
I	B0.56C0N	BFRP bars rupturing	13.50	51.85	32.23
	B0.77C0N	BFRP bars rupturing	14.10	73.28	35.23
	B1.15C0N	Concrete crushing	14.25	101.34	44.32
	B1.65C0N	Concrete crushing	15.00	101.43	46.83
II	B0.56C0E	BFRP bars rupturing	10.65	43.80	20.06
	B0.77C0E	BFRP bars rupturing	11.42	56.55	26.45
	B0.77C0.02E	BFRP bars rupturing	—	56.49	25.98
	B0.77C0.2E	BFRP bars rupturing	—	44.43	19.79
	B0.77C0.4E	BFRP bars rupturing	—	39.18	20.20
	B1.15C0E	BFRP bars rupturing	12.18	77.01	34.27
	B1.65C0E	BFRP bars rupturing	14.10	98.22	32.46
Average value			13.15	67.60	30.71
Standard deviations			1.55	23.97	9.31

**Table 6:** Experimental and theoretical results of the deflection of all beams when the stroke of the actuator reached 6 mm.

Beams	F_t_ (kN)	Δ_t_ (mm)	Δ_ACI15_ (mm)	Δ_ACI15_ /Δ_t_	Δ_ACI03_ (mm)	Δ_ACI03_ /Δ_t_	Δ_Ammash_ (mm)	Δ_Ammash_ /Δ_t_	Δ_c_ (mm)	Δ_c_ /Δ_t_
B0.56C0N	77.33	5.56	6.75	1.21	5.78	0.96	6.52	1.08	5.47	0.98
B0.77C0N	86.01	5.62	8.23	1.47	5.71	0.96	6.10	1.02	5.90	1.05
B1.15C0N	87.1	4.64	4.67	1.01	6.56	1.23	7.55	1.41	4.84	1.04
B1.65C0N	93.31	4.62	5.03	1.09	7.82	1.37	9.49	1.67	4.52	0.98
B0.56C0E	80.12	6.02	9.61	1.59	7.67	1.33	9.05	1.57	6.00	1.00
B0.77C0E	86.81	5.97	7.97	1.34	6.43	1.10	8.10	1.39	5.90	0.99
B0.77C0.02E	86.71	5.35	8.69	1.63	6.47	1.22	8.57	1.62	5.90	1.10
B0.77C0.2E	86.80	5.70	9.79	1.72	3.75	0.67	5.68	1.02	6.36	1.12
B0.77C0.4E	79.70	5.77	9.71	1.68	5.66	1.01	7.67	1.37	5.97	1.04
B1.15C0E	100.40	5.85	7.05	1.21	4.23	0.91	4.28	0.92	5.77	0.99
B1.65C0E	114.29	5.29	6.51	1.23	6.85	1.48	6.47	1.40	5.64	1.07
Average value				1.38		1.11		1.32		1.03
Standard deviations				0.25		0.24		0.26		0.05

## Data Availability

Not applicable.
